# The Role of Receptor for Advanced Glycation End Products (RAGE) in the Proliferation of Hepatocellular Carcinoma

**DOI:** 10.3390/ijms13055982

**Published:** 2012-05-18

**Authors:** Al-Madhagi Yaser, Yan Huang, Rong-Rong Zhou, Guan-Sheng Hu, Mei-Fang Xiao, Zhe-Bing Huang, Chao-Jun Duan, Wei Tian, Dao-Lin Tang, Xue-Gong Fan

**Affiliations:** 1 Department of Infectious Diseases, Xiangya Hospital, Central South University, Changsha 410008, China; E-Mails: yassnow@hotmail.com (A.-M.Y.); morninghy@yahoo.com.cn (Y.H.); rr_xy@hotmail.com (R.-R.Z.); huguansheng@126.com (G.-S.H.); xmf427@126.com (M.-F.X.); huangabing0330@yahoo.com.cn (Z.-B.H.); 2 Medical Science Institute, Xiangya Hospital, Central South University, Changsha 410008, China; E-Mail: duancjxy@126.com; 3 Immunogenetics Research Group, Department of Immunology, College of Basic Medical Sciences Central South University, Changsha 410013, China; E-Mail: tianwei3@yahoo.com; 4 Department of Surgery, Hillman Cancer Center, University of Pittsburgh Cancer Institute, Pittsburgh, PA 15219, USA; E-Mail: tangd2@upmc.edu

**Keywords:** RAGE, HMGB1, siRNA, NF-κB, proliferation

## Abstract

The receptor for advanced glycation end products (RAGE) is oncogenic and overexpressed in human cancers, but its role in hepatocellular carcinoma remains unclear. Here we demonstrated that RAGE is overexpressed in primary hepatocellular carcinoma (PHC) compared to adjacent para-neoplastic liver samples. Serum endogenous secretory RAGE levels were also increased in PHC patients (*p* < 0.01). Moreover, we demonstrated that RAGE regulates cellular proliferation in Hepatocellular carcinoma (HCC). Knockdown of RAGE by specific siRNA inhibited cellular growth in the hepatocellular carcinoma cell line, Huh7, whereas the RAGE ligand, high mobility group box 1 protein (HMGB1) increased cellular proliferation. In addition, knockdown of RAGE by siRNA arrested cells in the G1 phase and inhibited DNA synthesis (*p* < 0.01), while HMGB1 protein decreased the number of cells in the G1 phase and increased the number in the S phase (*p* < 0.05). Furthermore, quantitative real time RT-PCR (qRT-PCR) and Western Blot results demonstrated that RAGE and HMGB1 positively regulate NF-κB p65 expression in Huh7 cells. These studies suggest that RAGE and RAGE ligands are important targets for therapeutic intervention in hepatocellular carcinoma.

## 1. Introduction

Hepatocellular carcinoma (HCC) is one of the most common malignancies in the world, especially in Asia and Africa [1]. Most cases of HCC are secondary to either a viral hepatitis infection (hepatitis B or C) or cirrhosis [2]. Despite advances in surgical and nonsurgical therapies for hepatocellular cancer, there is still no satisfactory method for improving the overall survival rate of affected patients [3]. Since HCC is typically an inflammation-associated cancer, studying the molecular mechanisms underlying cancer promotion could be of great benefit to prevent hepatocarcinogenesis.

The receptors for advanced glycation end products (RAGE) are members of the immunoglobulin family which are usually expressed in low levels on a variety of cell types including immune cells, neurons, activated endothelial and vascular smooth muscle cells and bone forming cells. Physiologically, RAGE seems to have a role in embryonic neuronal outgrowth [4]. However, in adult, RAGE appears to act primarily in pathological responses including diabetes, inflammation, neuronal degeneration and cancers [5–8].

RAGE ligands fall into several distinct families. They include the High Mobility Group family proteins (including the HMGB1); members of the S100 protein family, and some advanced glycation end products such as carboxymethyllysine (CMLAGE) [9–12].

HMGB1 is a member of the non-histone, chromatin-associated high mobility group family of proteins [13]. It is widely expressed in many tumor cells and can be secreted by them or be released upon necrotic cell death [14,15]. The constant release of HMGB1, which functions as a proinflammatory cytokine, from necrotic tumor cells creates a microenvironment similar to chronic inflammation; a condition known to contribute to the development of epithelial malignancies, particularly inflammation-associated cancer [16–18].

The interactions between RAGE and its ligand trigger the activation of key cell signaling pathways (e.g., p38 and p44/42 MAP kinase [19], NF-κB, cdc42/rac [20]), and the generation of reactive oxygen species, and result in the production of pro-inflammatory cytokines [18]. NF-κB is a ubiquitous factor that controls the expression of genes involved in immune response and inflammation [21]. Its activity is regulated by homo- or hetero-dimers formed by members of the Rel/NF-κB family of transcription factors. The functional specificity and selectivity of the NF-κB response is thought to arise primarily from the binding of Rel/NF-κB complexes to specific DNA regulatory sites (κB sites) of target genes in various cell types [22,23].

To characterize the role of RAGE in the proliferation of HCC, we investigated RAGE expression in primary hepatocellular carcinoma (PHC) neoplastic and para-neoplastic liver samples. We also cultured the HCC cell line, Huh7, and diminished expression of RAGE using RAGE specific siRNA. Additionally, we activated RAGE using HMGB1. We also used RAGE siRNA and HMGB1 to explore the role of RAGE on the expression of the transcriptional factor NF-κB p65.

## 2. Results and Discussion

### 2.1. RAGE Expression in PHC Neoplastic Liver Samples Is Higher than Para-neoplastic Samples

**Figure 1:**
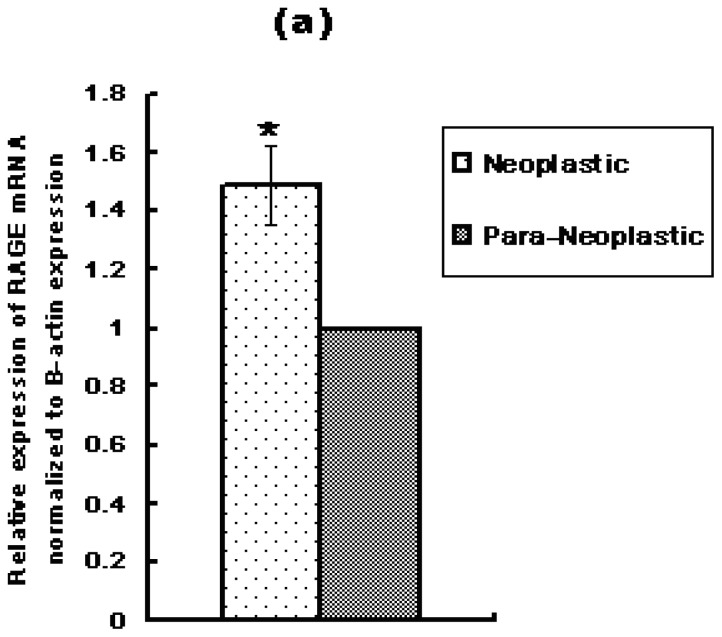

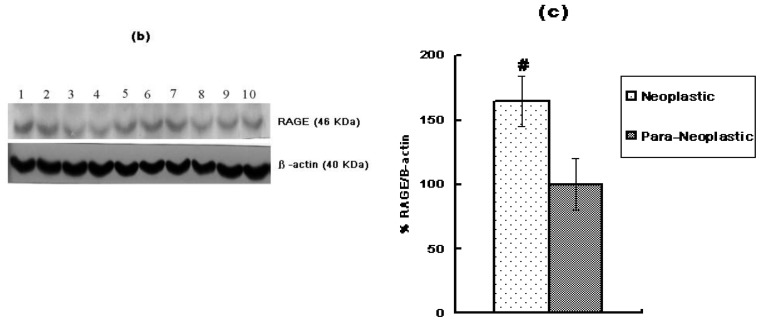
Expression of RAGE mRNA and protein in Primary Cellular Carcinoma (PHC) and their matched-adjacent liver tissues by qRT-PCR and Western Blot. (**a**) Relative expression of RAGE mRNA normalized to β-actin expression (neoplastic *vs.* para-neoplastic) analyzed by qRT-PCR (average ΔCt of ten samples); (**b**) Expression of RAGE protein by Western Blot; (**c**) Expression of RAGE protein as a ratio to β-actin. The pictures represent the results for 5 patients out of the total 10 patients. In all the patients RAGE mRNA and protein expression in PHC tissues was significantly higher than their matched-adjacent tissues (* *p* < 0.01, # *p* < 0.01). Lanes 2, 4, 6, 8, 10: RAGE expression in PHC. Lanes 1, 3, 5, 7, 9: RAGE expression in matched-adjacent liver tissues. M: molecular weight marker. Three independent measurements were made and averaged.

RAGE is overexpressed in a variety of human tumors. To determine whether RAGE is overexpressed in primary hepatocellular carcinoma, all tissue samples were ground and the cellular extracts or protein lysates were prepared and subjected to quantitative real time PCR (qRT-PCR) and Western Blot analysis. RAGE mRNA and protein expression in paired PHC neoplastic and para-neoplastic liver samples of the ten patients, demonstrated that PHC neoplastic liver samples express significantly higher levels of RAGE mRNA and protein than their para-neoplastic paired samples (*p* < 0.01), Figure 1. qRT-PCR results showed that RAGE mRNA expression in each neoplastic liver sample was significantly higher than its adjacent para-neoplastic sample (*p* < 0.01). Quantification analysis revealed that the average of RAGE mRNA expression in neoplastic samples was 50% higher than para-neoplastic samples. There was no significant difference in the level of RAGE expression between males and females.

### 2.2. RAGE Protein Level in the Sera of PHC Patients Is Higher than That in Controls

ELISA results demonstrated that RAGE is expressed in the sera of both PHC patients and normal control subjects. RAGE protein level in the sera of PHC patients was significantly higher than that in normal control subjects (313.34 ± 22.14 ng/L (*n* = 10), *vs.* 59.14 ± 4.21 ng/L (*n* = 10), *p* < 0.01). There was no significant difference in the level of serum RAGE between males and females.

### 2.3. RAGE siRNA Decreases Viability in Huh7 Cells, While HMGB1 Increases It

**Figure 2:**
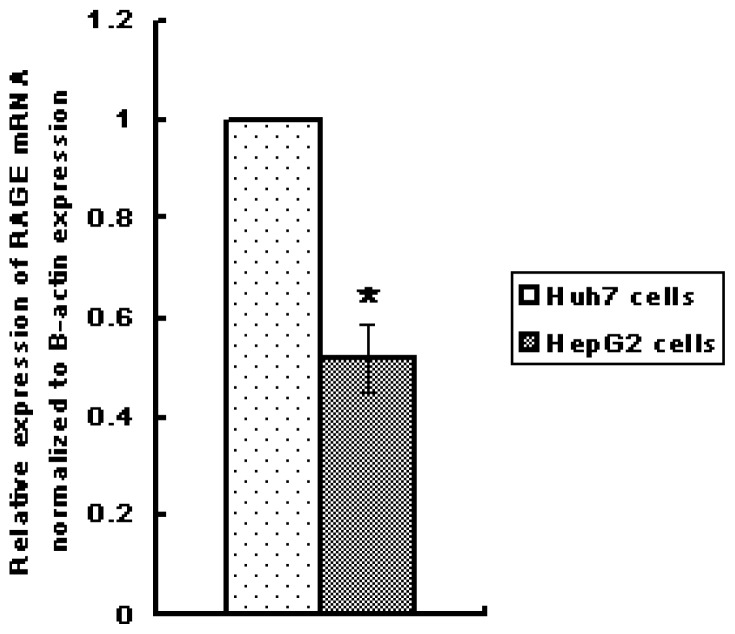
Relative expression of RAGE mRNA (normalized to β-actin expression) by HepG2 cells (*vs.* Huh7 cells) analyzed by qRT-PCR; (* *p* < 0.01). Three independent measurements were made and averaged.

Huh7 and HepG2 cells were cultured and expression of RAGE mRNA by qRT-PCR was evaluated. The results indicated that both Huh7 and HepG2 cells express RAGE mRNA, however, Huh7 cells express significantly higher levels of RAGE mRNA (*p* < 0.01), (Figure 2). According to this result, Huh7 was selected to undergo additional investigations.

**Figure 3:**
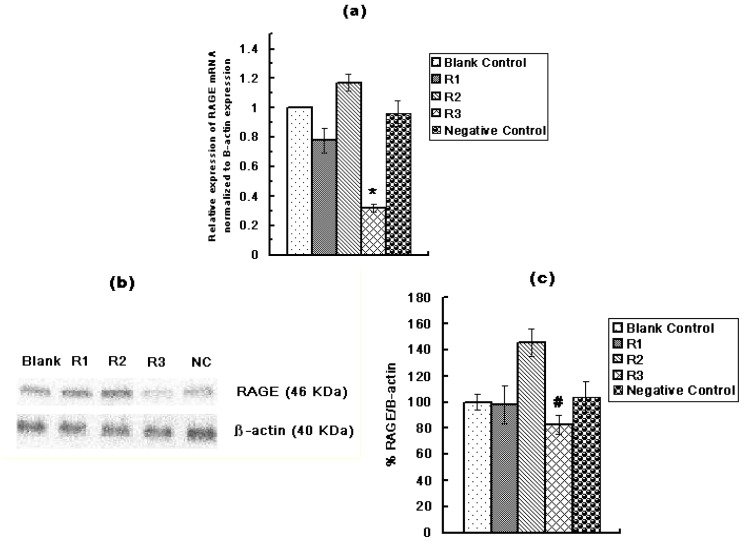
Expression of RAGE mRNA in Huh7 cells after transfection with three different RAGE siRNA; R1, R2, R3 and one negative control RNA (NC). (**a**) Relative expression of RAGE mRNA normalized to β-actin expression (*vs.* blank control) analyzed by qRT-PCR; (**b**) Expression of RAGE protein by Western Blot; (**c**) Expression of RAGE protein as a ratio to β-actin. R3 showed the highest efficiency in inhibiting RAGE gene products (* *p* < 0.01, # *p* < 0.01). Three independent measurements were made and averaged.

Huh7 cells were transfected with R1, R2, R3 or negative control RNA. qRT-PCR and Western Blot results demonstrated that R3 had the highest efficiency in inhibiting RAGE gene products (Figure 3). Therefore, R3 was selected as the silencing RNA to transfect Huh7 cells.

**Figure 4:**
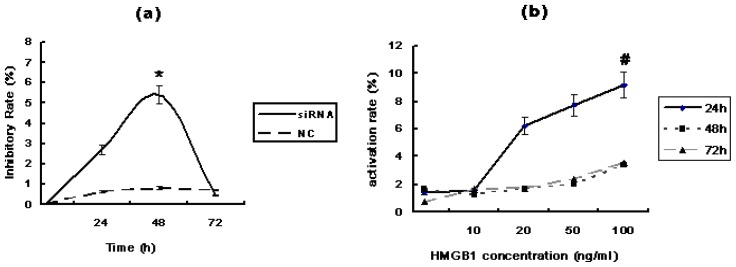
Cell proliferation was performed with the 3-(4,5-dimethylthiazol-2-yl)-2,5- diphenyltetrazolium bromide (MTT) colorimetric assay in Huh7 cells. (**a**) Cell growth inhibition caused by RAGE siRNA. The greatest growth inhibition was achieved after 48 h of incubation (* *p* < 0.01); (**b**) Cell growth activation caused by HMGB1. The greatest growth activation was observed after 24 h of incubation (# *p* < 0.01). Three independent measurements were made and averaged. NC: Negative Control RNA.

Thus, our results suggest that RAGE overexpression in PHC neoplastic liver samples and in the sera of PHC patients is closely associated with hepatocarcinogenesis. To explore whether RAGE contributes to HCC cellular proliferation, Huh7 cells were either transfected with RAGE siRNA or treated with HMGB1. MTT results indicated that Huh7 transfected with RAGE siRNA grew more slowly than cells transfected with negative control RNA (NC) and blank controls (*p* < 0.01). The greatest growth inhibition was achieved after 48 h of incubation. In contrast, HMGB1 activated the growth of Huh7 cells. The greatest increase in cell growth was observed after 24 h of incubation (*p* < 0.01), Figure 4.

### 2.4. RAGE siRNA Induces G1 Arrest in Huh7, While HMGB1 Induces DNA Synthesis

**Figure 5:**
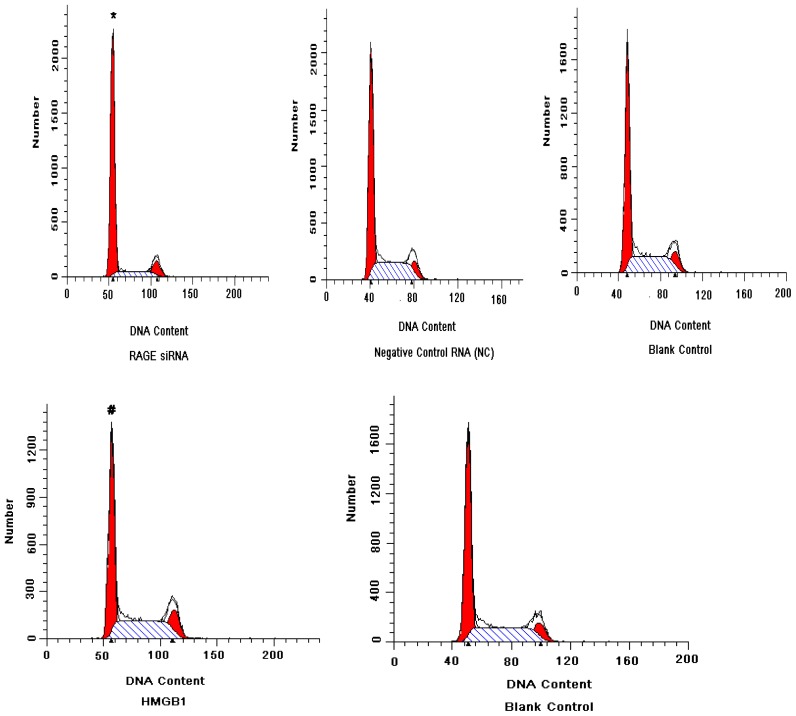
Cell cycle contribution of Huh7 detected by flow cytometry staining treated with RAGE siRNA or HMGB1. RAGE siRNA inhibited DNA synthesis, significantly increased the percentage of cells in G1 phase and significantly decreased the percentage of cells in S-phase 24 h post-treatment (* *p* < 0.01). However, HMGB1 induced DNA synthesis, significantly decreased the percentage of cells in G1 phase and significantly increased the percentage of cells in S-phase 24 h post-treatment (# *p* < 0.05). Three independent measurements were made and averaged.

MTT results demonstrated that RAGE is closely related to cellular proliferation. To confirm this result, we investigated the effect of RAGE siRNA and HMGB1 on the Huh7 cell cycle. FACS results indicated that growth inhibition by RAGE siRNA in the Huh7cells was accompanied by cell cycle G1 arrest, which was associated with a decrease in DNA synthesis and reduction in cellular growth. RAGE siRNA appears to inhibit DNA synthesis and increase the percentage of cells in G1 phase. After 24 h of treatment, the percentage of G1 phase cells was 75.59 ± 2.63% in the RAGE siRNA-treated group compared with 54.96 ± 3.47% in the negative control (NC) and 55.34 ± 3.22% in the blank control (*p* < 0.01). This increase was coupled with a decreased percentage of cells in S-phase. After 24 h of treatment, the percentage of S-phase cells was 14.10 ± 2.74% in the RAGE siRNA-treated group compared with 34.25 ± 3.37% in the negative control (NC) and 35.59 ± 3.93% in the blank control (*p* < 0.01). In contrast, HMGB1 induced DNA synthesis and decreased the percentage of cells in G1 phase. After 24 h, the percentage of G1 cells was 47.64 ± 3.21% in the HMGB1-treated group compared with 55.27 ± 2.86% in the blank control (*p* < 0.05). This decrease was coupled with an increased percentage of cells in S-phase. After 24 h of treatment, the percentage of cells in S-phase was 39.13 ± 3.57% in the HMGB1-treated group compared with 34.38 ± 2.43% in the blank control (*p* < 0.05), Figure 5.

### 2.5. RAGE siRNA Decreases the Expression of NF-κB p65 in Huh7 Cells, While HMGB1 Increases It

**Figure 6:**
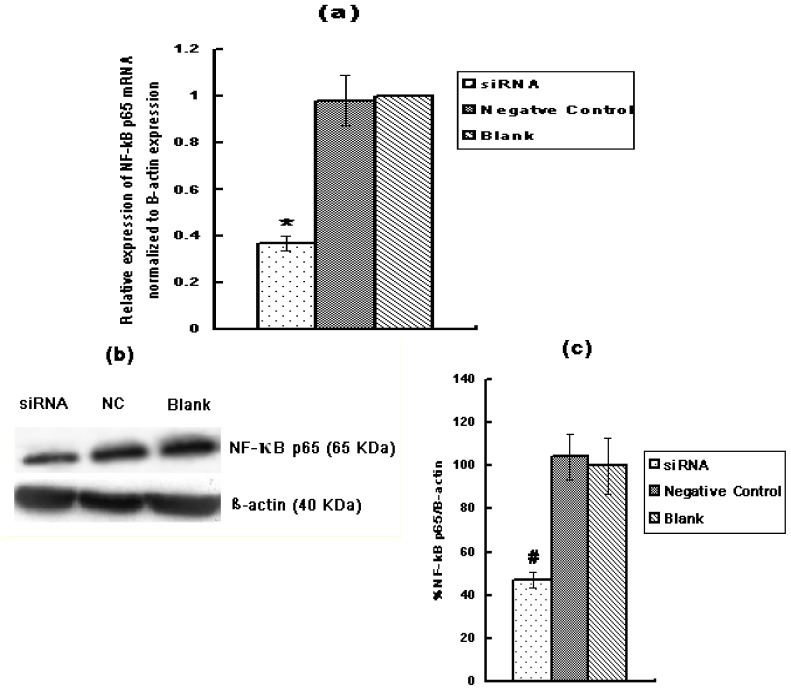
The effect of RAGE siRNA on the expression of NF-κB p65 in Huh7 cells by qRT-PCR and Western Blot. (**a**) Relative expression of NF-κB p65 mRNA normalized to β-actin expression (*vs.* blank control) analyzed by qRT-PCR; (**b**) Western Blot protein bands film results; (**c**) Expression of NF-κB p65 protein as a ratio to β-actin; (* *p* < 0.01, # *p* < 0.05). Three independent measurements were made and averaged. NC: Negative Control RNA.

**Figure 7:**
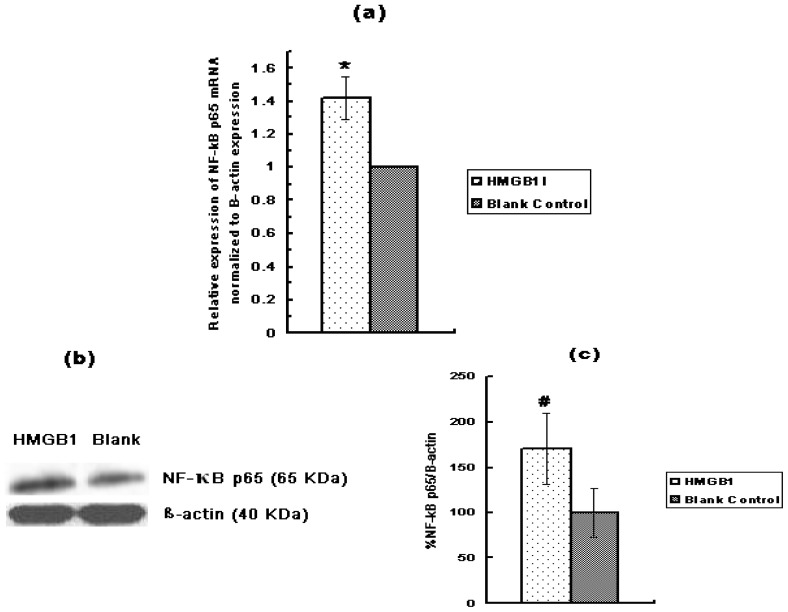
The effect of HMGB1 on the expression of NF-κB p65 in Huh7 cells by qRT-PCR and Western Blot. (**a**) Relative expression of NF-κB p65 mRNA normalized to β-actin expression (*vs.* blank control) analyzed by qRT-PCR; (**b**) Western Blot protein bands film results; (**c**) Expression of NF-κB p65 protein as a ratio to β-actin; (* *p* < 0.01, # *p* < 0.05). Three independent measurements were made and averaged.

NF-κB alters cell behavior in many ways. It inhibits apoptosis, increases cell proliferation, and increases inflammatory and immune response. Recent evidence suggests that activation of NF-κB contributes to the development of several types of human cancer. Accordingly, we investigated the role of RAGE siRNA and HMGB1 on Huh7 expression of NF-κB p65. Huh7 cells were either transfected with RAGE siRNA or treated with HMGB1. qRT-PCR and Western Blot results demonstrated that RAGE siRNA significantly decreased the expression of NF-κB p65 (Figure 6a,c), while HMGB1 significantly increased it (Figure 7a,c). qRT-PCR revealed that RAGE siRNA decreased the expression of NF-κB p65 by 68% (*p* < 0.01, *vs.* blank group, Figure 6a) while HMGB1 increased the expression of NF-κB p65 mRNA by 42% (*p* < 0.01, *vs.* blank group, Figure 7a).

### 2.6. Discussion

HCC is one of the most common forms of malignancy in humans worldwide, representing 40% of all the cancers in Southeast Asia, Japan, and Africa and 2%–3% of all the cancers in United States [24].

RAGE is expressed in a variety of human tumors, including ovarian, breast, colonic, brain, lung, prostate, lymphoma, and melanoma [25]. In most pathologic conditions in which it plays an important role, RAGE levels are found to be elevated [26–29]. Our results demonstrated a significant increase in RAGE expression in PHC liver samples comparing to their adjacent normal tissues. In addition, it showed for the first time a significant increase in RAGE serum levels in PHC patients compared to normal control subjects. Although the sample size in this part of the study was relatively small, the results gave us good introductory guidelines for our investigations on HCC cell lines which demonstrated that RAGE siRNA decreases Huh7 cellular proliferation, increases the number of Huh7 cells in the G1 phase and decreases their number in the S phase. This strongly correlates RAGE function to HCC growth and proliferation, and shows that inflammatory signals initiated by RAGE contribute greatly with hepatocarcinogenesis.

During our investigations on Huh7 cells, we chose the concentration of HMGB1 according to the results of MTT assay. The results demonstrated that 100 ng/mL of HMGB1 could significantly increase Huh7 cellular proliferation. Although Huh7 cells are expected to secrete HMGB1, it appears that adding exogenous HMGB1 amplifies the proinflammatory effect of the secreted HMGB1.

Promotion of cancer by HMGB1-RAGE interaction was demonstrated in various cancers, such as gastric cancer [30], colon cancer [31], breast cancer [26], melanoma [32], and prostate cancer [28]. On the other hand, previous studies showed that HMGB1 activates signaling pathway involving protein kinase B (AKT), mitogen-activated protein kinases (MAPKs), and NF-κB which play important roles in tumor growth [33]. Our results demonstrated that HMGB1 increases the expression of NF-κB p65, increases the number of cells in the S phase, and as a result increases the proliferation of Huh7 cells. This correlates HMGB1 to cellular proliferation, and completes the chain which starts with HMGB1 binding to RAGE, the activation of NF-κB and ultimately faster rate of cell proliferation and tumor growth.

In this study, we decreased the expression of RAGE through RAGE specific siRNA and found that NF-κB p65 expression was decreased. On the other hand, we activated RAGE using HMGB1 and found that expression of NF-κB p65 was increased. This correlates RAGE activity to NF-κB p65 expression and confirms that RAGE activation perpetuate NF-κB p65 activation by de novo synthesis of NF-κB p65. Thus this pathway produces a constantly increasing pool of NF-κB p65 which is directly associated with cell proliferation.

Experimental evidence demonstrated that NF-κB binding to cyclin D1 promoter is critical for the regulation of cyclin D1 expression [34]. This indicates that signal transduction begins with RAGE through NF-κB leading to enhanced expression of cyclin D1 which in turn leads to a rapid progression from G1 to S phase and an increased rate of division and proliferation of cancer cells. Moreover, the increase in RAGE expression is thought to be due to a positive feedback loop from the RAGE promoter through RAGE activation of NF-κB [35].

Taking together, we demonstrated that interfering with RAGE activity through the use of specific siRNA could be of great benefit in reducing the proliferation of hepatocellular carcinoma. This indicates that RAGE gene is a potential target for future gene therapy researches to find treatments for many malignancies, including HCC. In addition, several studies have addressed the identification of putative NF-κB inhibitors as therapeutic agents for cancer. Since NF-κB activation is the result of a multi-step signaling pathway, these agents may target different points of the signaling process. Other means to reduce cellular proliferation could be through the use of RAGE blocking antibodies which needs further investigations.

## 3. Experimental Section

### 3.1. Patients and Controls

This part of the study included 10 cases of primary hepatocellular carcinoma (PHC). Liver tumors in those patients were removed surgically in the department of General Surgery, Xiangya Hospital of Central South University, during the period June 2010–August 2010. The neoplastic, correspondent para-neoplastic tissues and blood samples of the patients were collected. All liver samples were independently reviewed and diagnosed by two pathologists. Exclusion criteria included patients with severe infection, sepsis, end-stage renal failure, diabetes, neurological disease, patients using immunomodulatory drugs and patients who underwent radiotherapy during past three months. Of the ten cases, seven were males and three were females, average age 46.7 ± 15.3 years. Other characteristics of the patients were presented in Table 1. During the same period of time, 10 blood samples were collected from healthy volunteers (7 males and 3 females); average age 41.6 ± 12.7 years, as a control. Written informed consent to use the samples for research was obtained from the patients and clinicians. The present study protocol was approved by the ethical committee at Xiangya Hospital of Central South University, Changsha, China.

#### ELISA

Blood samples were kept for 10–20 min to coagulate and then centrifuged at 3000 rpm for 20 min. Supernatants were collected and RAGE expression was analyzed by ELISA (Abcam, USA) according to the manufacturer instructions. Three independent measurements were made and averaged.

### 3.2. Cell Culture

Human hepatocellular carcinoma cell lines Huh7 and HepG2 were stored in our laboratory. Huh7 cells were cultured in RPMI 1640 medium (Invitrogen, USA), while HepG2 cells were cultured in Dulbecco’s modified essential medium DMEM (Invitrogen, USA). Both media were supplemented with 10% heat inactivated fetal bovine serum (Invitrogen, USA), 100 units/mL Ampicillin and 100 mg/mL of streptomycin. The cells were incubated at 37 °C in humidified incubator with 5% CO_2_.

### 3.3. Quantitative Real Time PCR

Tissue samples were cut and 30 mg of each sample was weighed and ground. To investigate the effect of RAGE siRNA, Huh7 cells were cultured in 6-well plate and divided into three groups; the siRNA group (transfected with RAGE siRNA), the negative control group (transfected with negative control RNA to prevent induction of nonspecific cellular events caused by introduction of the oligonucleotide into cells) and the blank control group. However, to investigate the effect of HMGB1, Huh7 cells were cultured in 6-well plate and divided into two groups, one group was treated with 100 ng/mL human recombinant HMGB1 (PROSPEC, USA), while the other group was kept as a blank control. After 24 h, culture media were removed and the cells were washed with PBS. Total RNA from the tissue samples and cell cultures was isolated with Trizol (Invitrogen, USA) and converted to cDNA by reverse transcription. qRT-PCR amplifications were performed with a PCR Mastercycler^®^ epgradient Eppendorf (Germany). Amplification was carried out in 20 μL reactions containing 0.8 μL of primer, 10 μL of Platinium SYPR Green qPCR SuperMix-UDG (Invitrogen, China), and 1 μL of cDNA. To compare treated group to blank group, the ΔCt of each group was calculated by formula: ΔCt_1_ = Ct_treated_ − Ct_β-actin_ and ΔCt_2_ = Ct_blank_ − Ct_β-actin_. ΔΔCt was calculated by (ΔCt_2_ − ΔCt_1_). The fold-change for gene expression levels of the treated groups were calculated using 2ΔΔCt. To compare neoplastic to para-neoplastic, the ΔCt was calculated by formula: ΔCt_1_ = Ct_neoplastic_ − Ct_β-actin_ and ΔCt_2_ = Ct_para-neoplastic_ − Ct_β-actin_. ΔΔCt was calculated by (ΔCt_2_ − ΔCt_1_). The fold-change for gene expression levels of neoplastic group was calculated by formula 2ΔΔCt.

The primer sequences used for RAGE qRT-PCR were: 5′-GGCTGGTGTTCCCAATAAGG-3′ and 3′-TCACAGGTCAGGGTTACGGTTC-5′.

NF-κB P65 primer sequences were: 5′-TGCTGTGCGGCTCTGCTTCC-3′ and 3′-AGGCTC GGGTCTGCGTAGGG-5′.

β-actin was used as a control for RNA integrity with the following primers sequences: 5′-GACAGGATGCAGAAGGAGATTACT-3′ and 3′-TGATCCACATCTGCTGGAAGGT-5′.

### 3.4. Western Blot

Tissue samples were cut and 10 g of each sample was weighed and ground. Huh7 cells were cultured and either transfected with RAGE siRNA or treated with HMGB1 as described in Section 3.3. After 48 h of incubation, culture media were removed and the cells were washed with PBS. Total protein from tissue samples and cell cultures was extracted by RIPA buffer (Beytime, China). After that, the protein amount was measured by BCA method (Beytime, China). 40 μg of the total protein was loaded and separated on a 12% Acrylamide/Bis gel (Beytime, China) and the protein was transferred to a PVDF membrane. The PVDF membrane was blocked with 5% Bovine Serum Albumin (BSA) in TBST. RAGE mouse monoclonal antibody (Santa-Cruz, USA) was diluted in BSA/TBST solution (1:500) and used to detect RAGE protein in the tissue samples. NF-κB p65 mouse monoclonal antibody (Santa-Cruz, USA) was diluted in BSA/TBST solution (1:500) and used to detect NF-κB p65 protein in cell cultures. The PVDF membrane was incubated with the antibody for 30 min at room temperature and over night at 4 °C and then washed three times (10 min each) with TBST. After that, the PVDF membrane was incubated with HRP-labeled mouse IgG secondary antibody (Santa-Cruz, USA) diluted in TBST (1:1000) for 1 h and then washed three times (10 min each) with TBST. Protein bands were examined by ECL chemiliumenescent reagents (Beytime, China). Band intensities were measured and protein signals were normalized with β-actin.

### 3.5. Transfection and Selection of siRNA

Three siRNA (R1, R2 and R3) and one negative control (NC) RNA were synthesized by GenePharma Company (Shanghai, China). The sequences of the RNAs were as follow:

R1: 5′-CACUGGUGCUGAAGUGUAATT-3′
5′-UUACACUUCAGCACCAGUGTT-3′
R2: 5′-CUCCUCAAAUCCACUGGAUTT-3′
5′-AUCCAGUGGAUUUGAGGAGTT-3′
R3: 5′-GACCAACUCUCUCCUGUAUTT-3′
5′-AUACAGGAGAGAGUUGGUCTT-3′
NC: 5′-UUCUCCGAACGUGUCACGUTT-3′
5′-ACGUGACACGUUCGGAGAATT-3′

Huh7 Cells in logarithmic growth phase were collected and dispensed in 6-well culture plate in 2 mL volumes. After 12 h, cells were transfected independently with R1, R2, R3 or the negative control RNA (NC) as follow: 100 pmol of the siRNA was added into 250 μL of Opti-MEM Reduced-Serum Medium (Invitrogen, USA) and mixed gently. At the same time, 5 μL of Lipofectamin (Invitrogen, USA) was added into 250 μL of Opti-MEM. After 5 min the Opti-MEM including siRNA was mixed gently with the Opti-MEM including the Lipofectamin. After 20 min the mixture was added into the wells and plates were incubated at 37 °C and 5% CO_2_ for 24 h.

### 3.6. MTT Assay

Cell proliferation assay was performed with 3-(4,5-dimethylthiazol-2-yl)-2,5-diphenyltetrazolium bromide (MTT). Briefly, Huh7 cells in logarithmeic growth phase were collected and dispensed in 96-well culture plate in 200 μL volumes. The cells were cultured and either transfected with RAGE siRNA or treated with HMGB1 as described in Section 3.3. The cells were then incubated at 37 °C and 5% CO_2_ for 0, 24, 48, and 72 h. The MTT solution was prepared by mixing 5 mg of MTT powder with 10 mL PBS, and sterilized by passing through 0.22 μm filter unit. At the end of each indicated incubation interval, the cells were washed with PBS buffer and 50 μL of the MTT solution was added into each well. The plates were incubated for 4 h at 37 °C and 5% CO_2_. Then, the MTT solution was removed and 150 μL of DMSO was added into each well. Finally, the absorbance (A value) was measured using a micro-culture plate reader at 490 nm. Cell proliferation inhibition rate by RAGE siRNA was calculated according to the following equation: inhibition rate = [(absorbance value of blank control group at 490 nm) − (absorbance value of the transfected group at 490 nm)/absorbance value of blank control group at 490 nm] × 100. The increase in cells growth caused by HMGB1 was referred to as “the activation rate” and was calculated according to the following equation: activation rate = [(absorbance value of HMGB1 group at 490 nm) − (absorbance value of blank control group at 490 nm)/absorbance value of blank control group at 490 nm] × 100.

### 3.7. Cell Cycle Analysis

Huh7 cells were cultured into 6-well culture plates and either transfected with RAGE siRNA or treated with HMGB1 for 24 h as described in Section 3.3. Treated cells were harvested with trypsin, then washed once with 4 °C PBS and fixed in cold 75% ethanol at 4 °C. Cells were then washed once again with 4 °C PBS and re-suspended with PBS, then stained with 10 μg/mL propidium iodide (Beijing Dingguo, China) and 0.5 mg/mL RNase A solution (Fermentas, EU) for 30 min at 37 °C in dark. Stained cells were immediately subjected to analysis by flow cytometry. The proportion of cells in each phase of the cell cycle was determined by a FACScan for Quantitative Cell Analysis.

### 3.8. Statistical Analysis

ImageJ software was used to measure the size and percentage of DNA and protein bands on the gels and films. All data were analyzed using SPSS 11.5 software package for Windows and were summarized as mean ± SE. One-way ANOVA was used to compare the data among three or more groups; Student’s *t*-test was used to compare two groups. For all tests, a two-sided probability (*p* < 0.05) was considered statistically significant.

## 4. Conclusions

We demonstrated that RAGE regulates the proliferation of hepatocellular carcinoma. Inflammatory signals initiated by RAGE contribute greatly to hepatocarcinogenesis. This indicates that RAGE gene could be a potential target for future gene therapy researches to find treatments for many malignancies, including HCC. Another means of reducing cellular proliferation could be through the use of NF-κB inhibitors or RAGE specific antibodies; however, this requires further investigations.

## Figures and Tables

**Table 1:** Clinical characteristics of PHC patients enrolled in the study

Number of patients	10
Male/Female	7/3
Mean age (average)	46.7 ± 15.3 years
HBsAg (+)	8
HBV-DNA	8 (10^2^ ~ 10^6^ copies/ml)
Anti-HCV (+)	2
HCV-RNA (+)	1 (10^5^ copies/ml)
Cirrhosis	7
